# Clinical impact of post‐progression survival on overall survival in patients receiving nivolumab monotherapy as a second‐line treatment for advanced non‐small cell lung cancer

**DOI:** 10.1111/1759-7714.13886

**Published:** 2021-02-24

**Authors:** Hisao Imai, Ou Yamaguchi, Keita Mori, Kosuke Hashimoto, Tomoe Akagami, Shun Shinomiya, Yu Miura, Ayako Shiono, Atsuto Mouri, Kyoichi Kaira, Kunihiko Kobayashi, Hiroshi Kagamu

**Affiliations:** ^1^ Department of Respiratory Medicine, Comprehensive Cancer Center, International Medical Center Saitama Medical University Saitama Japan; ^2^ Clinical Research Support Center Shizuoka Cancer Center Suntou‐gun Japan

**Keywords:** nivolumab, non‐small cell lung cancer, survival

## Abstract

**Background:**

The effect of second‐line treatment on overall survival (OS) may be affected by subsequent treatment in patients with non‐small cell lung cancer (NSCLC); however, in such patients, the correlation between post‐progression survival (PPS) and OS is unclear. Our study assessed the correlation of progression‐free survival (PFS) and PPS with OS, using individual patient data, in advanced NSCLC patients who were treated with second‐line nivolumab monotherapy,

**Methods:**

Between January 2016 and March 2019, we evaluated 92 NSCLC patients who received second‐line nivolumab treatment after first‐line platinum‐based combination chemotherapy. Using individual patient data, the correlations of PFS and PPS with OS were examined.

**Results:**

Linear regression and Spearman rank correlation analysis demonstrated that PPS was strongly correlated with OS (*r* = 0.85, *p* < 0.05, *R* ^*2*^ = 0.75), while PFS was moderately correlated with OS (*r* = 0.65, *p* < 0.05, *R* ^*2*^ = 0.42). Performance status at the beginning of second‐line treatment, immune checkpoint inhibitor rechallenge, and the number of treatment regimens used post‐progression, after the second‐line treatment significantly correlated with PPS (*p* < 0.05). In advanced NSCLC patients who underwent second‐line treatment with nivolumab, in comparison to PFS, there was a stronger correlation between PPS and OS.

**Conclusions:**

Our findings suggest that subsequent treatment for disease progression after a second‐line nivolumab treatment had a significant impact on OS.

## Graphical abstract

Post‐progression survival (PPS) influences overall survival (OS) in NSCLC patients who receive second‐line nivolumab monotherapy. When compared with PFS, PPS was more strongly correlated with OS. Subsequent treatment after disease progression following second‐line nivolumab treatment had a significant impact on OS.

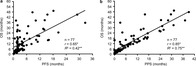

## INTRODUCTION

Non‐small cell lung cancer (NSCLC) constitutes 85%–90% of lung cancer cases.^1^ Overall survival (OS) is regarded as the most reliable and favorable endpoint in oncology clinical trials.^2^ OS is accurate and can be conveniently recorded by the date of death. Alternative endpoints for phase II trials include progression‐free survival (PFS) and overall response. These endpoints are more accessible and can be monitored sooner as the related events tend to occur frequently and at an earlier date (vs. the date of death for calculating OS).

With the increasing availability of different chemotherapy regimens for lung cancer, the impact of first‐line treatment on OS may be affected by subsequent treatments.^3^ A phase III randomized trial has shown that a favorable PFS does not necessarily translate into an OS advantage in patients with NSCLC.^4^ As with the management of breast, colorectal, and ovarian malignancies,^5^, ^6^, ^7^ an increasing choice of available drugs has allowed the development of subsequent‐line treatment for advanced or metastatic NSCLC. For first‐line treatment, PFS has not yet been established as an alternative endpoint for OS. Clinical studies have found a strong correlation between post‐progression survival (PPS) and OS, following first‐, second‐, and third‐line treatment, in patients with advanced or metastatic NSCLC.^8^, ^9^, ^10^ Furthermore, in patients with advanced or metastatic NSCLC, PPS has been found to correlate strongly with OS, particularly since the introduction of molecular targeted drugs, including epidermal growth factor receptor, tyrosine kinase inhibitors such as gefitinib and erlotinib, in the 2000s.^8^, ^9^ A simple and convenient tool for rating PPS, where OS is defined as PFS plus PPS, has been described.^2^ A review article^11^ reported that in patients who underwent systemic chemotherapy for lung cancer, PPS, rather than PFS, was more strongly correlated with OS, possibly because of the availability of intensive subsequent treatment.

The impact of cancer treatment on survival in patients who underwent treatment post‐progression at the individual patient level is unclear. Previous studies^12^, ^13^, ^14^ evaluating individual patient data have shown a strong relationship between PPS and OS after first‐line therapy in patients with NSCLC or small cell lung cancer (SCLC). Similarly, recent studies^15^, ^16^ have demonstrated that after second‐line systemic chemotherapy in NSCLC patients, PPS was closely associated with OS at the individual patient level. However, in metastatic NSCLC patients receiving second‐line nivolumab monotherapy, the correlation between PPS and OS is unclear. Therefore, it might be clinically relevant to use individual patient data to determine whether PFS or PPS is more closely associated with OS in metastatic NSCLC patients who underwent second‐line nivolumab monotherapy.

Recent clinical trials^17^, ^18^, ^19^, ^20^ have found that, following the failure of platinum combination chemotherapy in patients with NSCLC, administration of programmed cell death‐1 or programmed cell death‐ligand‐1 blockade antibodies was associated with longer OS than standard chemotherapy. Immune checkpoint inhibitor (ICI) (nivolumab, pembrolizumab, or atezolizumab) therapy is now regarded as the standard subsequent‐line treatment in patients without contraindications to ICIs. Moreover, in patients with disease progression either during or after first‐line treatment, nivolumab monotherapy is the standard second‐line treatment. In clinical trials,^17^, ^18^ nivolumab monotherapy was administered in NSCLC patients with disease progression after one chemotherapy regimen.

Although several phase III oncology trials have investigated previously treated patients with advanced or metastatic NSCLC,^15^, ^16^ only a few studies, evaluating PPS for this patient cohort, are based on individual patient data. Therefore, in this study we aimed to evaluate the correlation of PFS and PPS with OS in patients who received second‐line nivolumab monotherapy for advanced NSCLC and determine how subsequent treatments may influence PPS using individual patient data.

## METHODS

### Ethics statement

This study was approved by the ethics committee of the Saitama Medical University International Medical Center (approval number: 20‐137). All procedures involving human participants were performed in accordance with the ethical standards of the institutional and/or national research committee and with the 1964 Helsinki Declaration, as well as its later amendments or comparable ethical standards. The requirement for written informed consent was waived by the ethics committee of Saitama Medical University International Medical Center owing to the retrospective nature of the study.

### Patients

**FIGURE 1:**
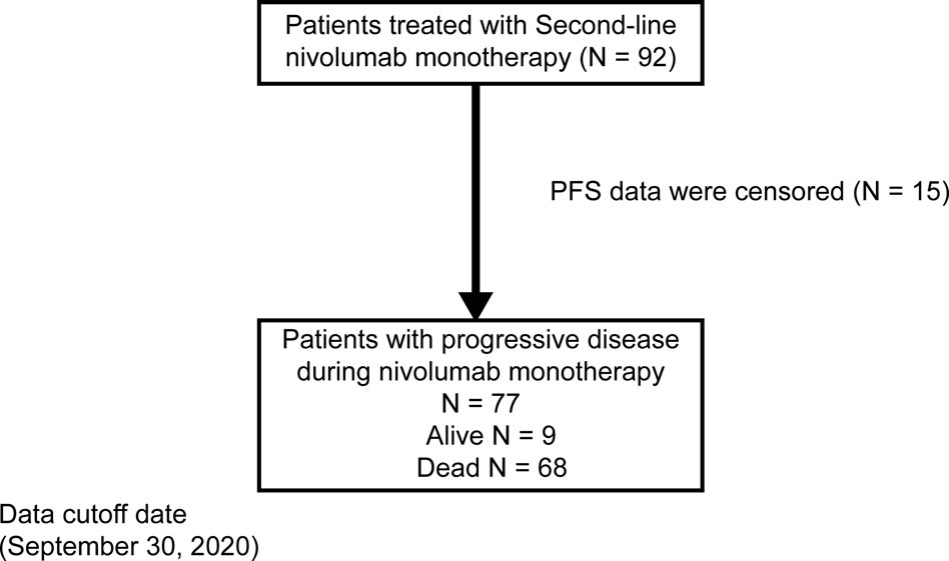
Flow chart showing patient selection. Patients received nivolumab monotherapy as a second‐line treatment between January 2016 and March 2019. PFS, progression‐free survival

In this study, 92 consecutive patients with advanced or metastatic NSCLC who received first‐line platinum‐based combination chemotherapy and second‐line nivolumab monotherapy between January 2016 and March 2019 were included. Of these, 77 patients with disease progression after the administration of second‐line nivolumab monotherapy were enrolled in the final analysis (Figure 1). Nivolumab monotherapy was administered in these patients at the Comprehensive Cancer Center, Saitama Medical University International Medical Center, Japan. The inclusion criteria were as follows: (i) histologically or cytologically confirmed NSCLC, (ii) platinum‐based combination chemotherapy administered as first‐line treatment, (iii) nivolumab monotherapy administered as second‐line treatment, (iv) disease progression beyond second‐line nivolumab monotherapy, and (v) verification of censored event or death.

### Treatments

Nivolumab (3 mg/kg or 240 mg/day) was administered intravenously every two weeks; the drug was administered repeatedly until disease progression, unacceptable adverse events, and/or patient rejection. The first‐ and subsequent‐line treatments were administered by treating physicians.

### Assessment of treatment efficacy

Tumor response was quantified based on the best overall response and maximum tumor shrinkage. Radiological tumor response was assessed according to the Response Evaluation Criteria in Solid Tumors (version 1.1).^21^ Response based on target and nontarget lesions were defined as follows: complete response (CR), the disappearance of all target lesions; partial response (PR), a ≥30% decrease in the sum of the diameters of the target lesions relative to the baseline; progressive disease (PD), a ≥20% increase in the sum of the diameters of the target lesions relative to the smallest value observed during the study period; and stable disease (SD), a shrinkage insufficient to be qualified as PR and a growth insufficient to be qualified as PD. PFS was measured from the start of nivolumab administration until PD or death from any cause. OS was measured from the first day of nivolumab therapy until death or was censored at the last follow‐up. PPS was measured from the date of tumor progression after second‐line nivolumab monotherapy until death or censored at the last follow‐up.

### Statistical analysis

Linear regression analysis and Spearman's rank correlation coefficients were used to evaluate the association of PFS and/or PPS with OS. A Cox proportional hazards model with stepwise regression was used to identify factors that predicted PPS. The results were expressed as hazard ratios with 95% confidence intervals. Because the hazard ratio was defined for a 1‐unit difference, some factors were converted to an appropriate scale unit. PPS was compared using the log‐rank test. All statistical analyses were conducted using JMP software for Windows (version 11.0) (SAS Institute). Two‐tailed *p*‐values of ≤0.05 were considered statistically significant.

## RESULTS

### Patient backgrounds

**FIGURE 2:**
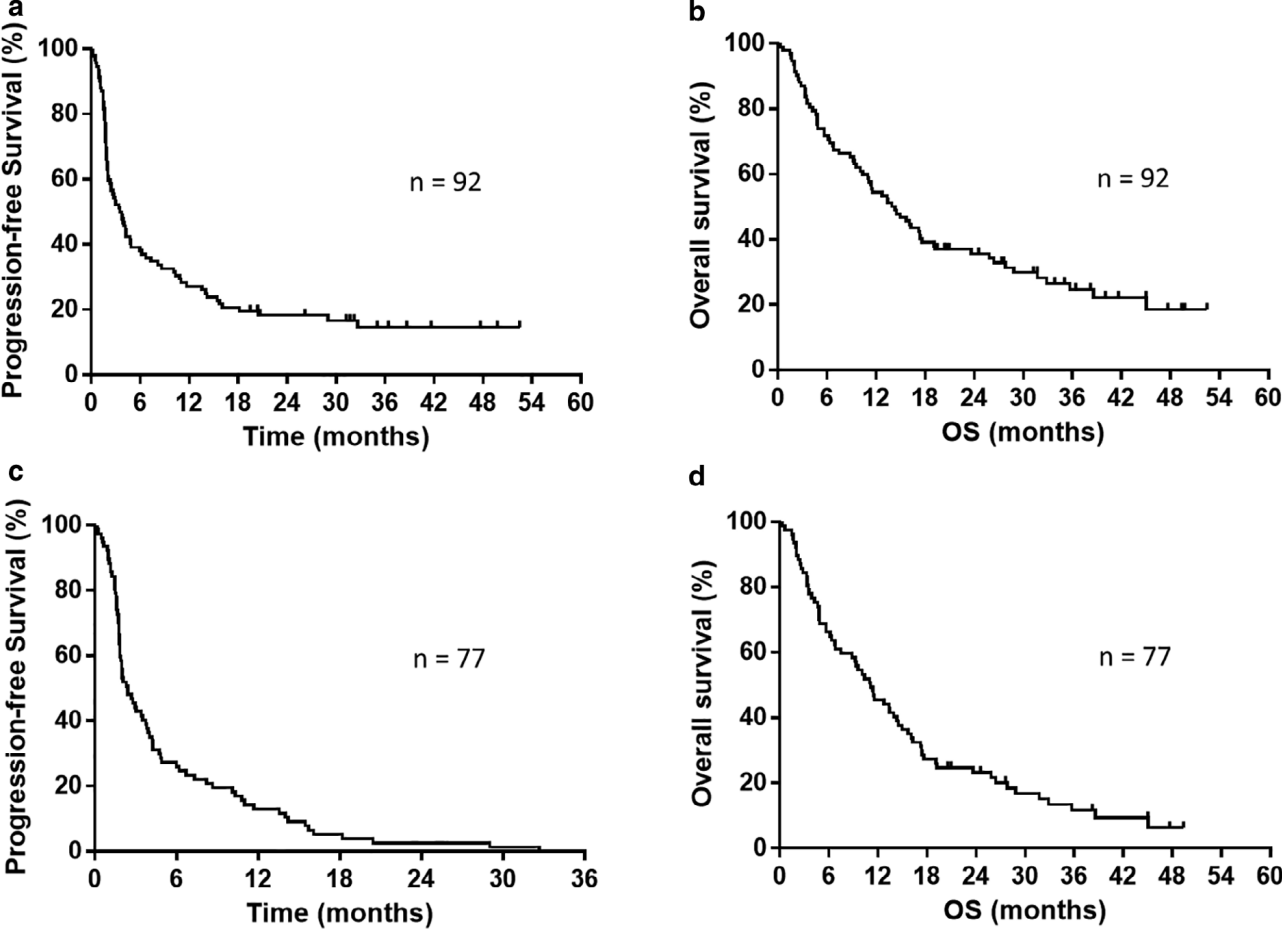
Kaplan–Meier curves of progression‐free survival (PFS) and overall survival (OS). (a) PFS from the start of second‐line nivolumab treatment (all 92 patients received nivolumab as second‐line treatment); median PFS from the start of second‐line chemotherapy: 3.5 months. (b) OS from the start of second‐line nivolumab treatment (all 92 patients received nivolumab as second‐line treatment); median OS: 14.1 months. (c) PFS from the start of second‐line chemotherapy (77 patients with disease progression following second‐line nivolumab monotherapy); median PFS from the start of second‐line nivolumab treatment: 2.4 months. (d) OS from the start of second‐line nivolumab treatment (77 patients with disease progression following second‐line nivolumab monotherapy); median OS: 11.1 months

Patient demographics (92 patients receiving second‐line nivolumab monotherapy and 77 patients with disease progression) are summarized in Table 1. Of the 92 patients who received nivolumab monotherapy, one, 19, 28, and 38 patients achieved CR, PR, SD, and PD, respectively. The overall response rate was 21.7%, and the disease control rate was 52.1%. The median PFS, from the start of second‐line nivolumab administration, was 3.5 months (Figure 2(a)). The median OS, from the start of second‐line nivolumab administration, was 14.1 months (Figure 2(b)). The median follow‐up duration was 14.1 (range, 0.3–52.4) months.

**TABLE 1:** Baseline patient characteristics at the beginning of second‐line treatment

Characteristics	Total (*N* = 92)	Progressive disease (*N* = 77)
Sex (*N*)		
Male/female	73/19	60/17
Age at treatment (years), median (range)	69 (31–79)	69 (31–79)
PS (*N*)		
0/1/2/3/4	37/39/10/6/0	28/33/10/6/0
Smoking history (*N*)		
Yes/no/unknown	80/12/0	66/11/0
Histology (*N*)		
Adenocarcinoma/squamous cell carcinoma/other	52/22/18	43/18/16
Clinical stage at diagnosis (*N*)		
III/IV/postoperative recurrence	16/59/17	13/52/12
PD‐L1 TPS (*N*)		
<1%/1%–49%/≥50%/unknown	13/5/1/73	10/3/1/63
Driver mutation/translocation (*N*)		
*EGFR*/*ALK*/*ROS‐1*/wild‐type/unknown	3/0/1/88	3/0/1/73
Platinum combination therapy as first‐line chemotherapy (*N*)		
CDDP‐based/CBDCA‐based	21/71	17/60
With bevacizumab/without bevacizumab	23/69	21/56
Therapeutic effect of first‐line chemotherapy (*N*)		
CR/PR/SD/PD/NE	1/39/35/17	1/30/31/15
Prior radiotherapy (including palliative intent) (*N*)		
Yes/no	35/57	28/49
ICI rechallenge (*N*)		
Yes/no	14/78	14/63
Number of cycles of nivolumab (*N*)		
Median (range)	4 (1–82)	4 (1–50)
Number of regimens after disease progression following second‐line nivolumab (*N*)		
0/1/2/3/4/5/≥6	—	36/21/11/5/2/1/1
Median (range)	—	1 (0–7)
Follow‐up period (months), median (range)	14.1 (0.3–52.5)	14.1 (0.3–49.4)

Abbreviations: CBDCA, carboplatin; CDDP, cisplatin; CR, complete response; ICI, immune checkpoint inhibitor; NE, not evaluated; PD, progressive disease; PD‐L1, programmed cell death‐ligand 1; PR, partial response; PS, performance status; SD, stable disease; TPS, tumor proportion score.

Of the 77 patients included in the analysis, 68 were dead and nine were alive at the data cutoff (September 30, 2020). The median follow‐up duration was 11.1 (range, 0.3–49.3) months. Of the 77 patients with disease progression, one, 12, 20, and 38 patients achieved CR, PR, SD, and PD, respectively. The overall response rate was 16.8%, and the disease control rate was 42.8%. In case of progression after second‐line treatment, 36 patients received only best supportive care; the median number of subsequent regimens was one (range, 0–7 regimens). The regimens used as subsequent chemotherapy, following second‐line treatment, are shown in Table 2. The median PFS from the start of second‐line treatment was 2.4 months (Figure 2(c)), and the median OS was 11.1 months (Figure 2(d)).

**TABLE 2:** Number of chemotherapy regimens used as subsequent chemotherapy following second‐line nivolumab

Treatment	Third‐line	≥Fourth‐line	Total
Single cytotoxic agents			
Docetaxel	9	0	9
Pemetrexed	1	4	5
S‐1	0	6	6
Other	0	2	2
Docetaxel plus ramucirumab	26	1	27
Platinum‐based combination	3	2	5
Non‐platinum‐based combination	0	1	1
Molecular targeted drug	0	9	9
ICI rechallenge	3	11	14
Investigational agent	0	1	1
Best supportive care	36	—	36

Abbreviation: ICI, immune checkpoint inhibitor.

### Correlations of PFS and PPS with OS

**FIGURE 3:**
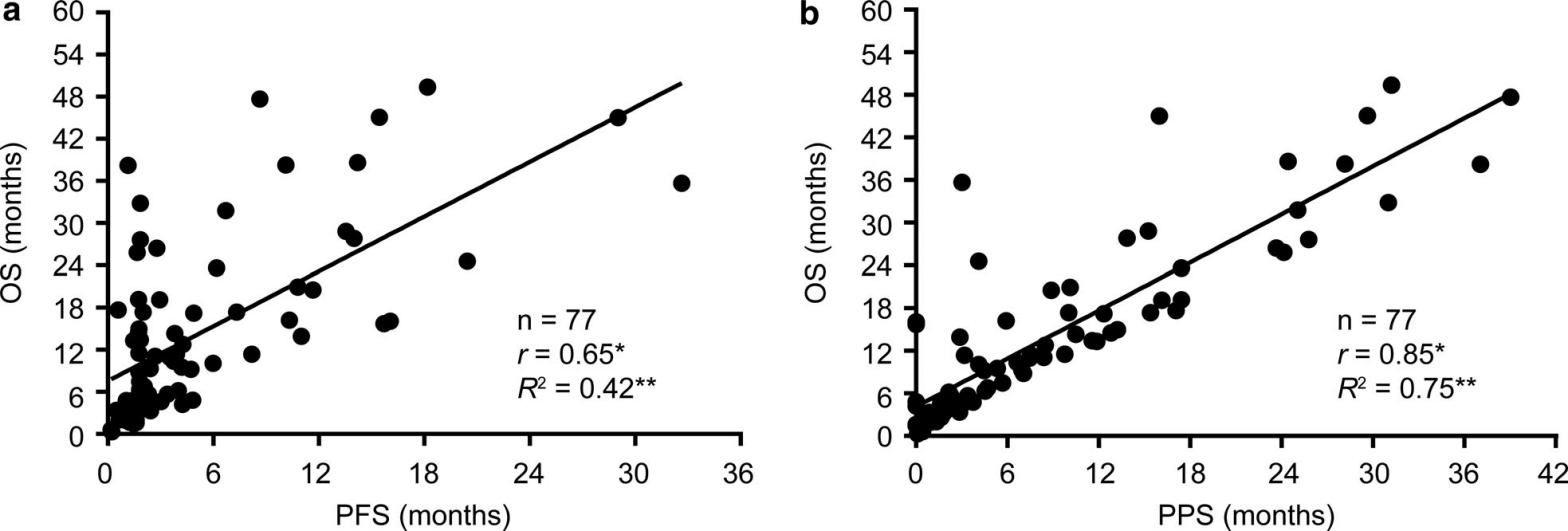
Correlation of progression‐free survival (PFS) and post‐progression survival (PPS) with overall survival (OS) from the start of second‐line nivolumab treatment. (a) Correlation between OS and PFS. (b) Correlation between OS and PPS. *r values represent Spearman's rank correlation coefficients. **R ^*2*^ values represent linear regression

**FIGURE 4:**
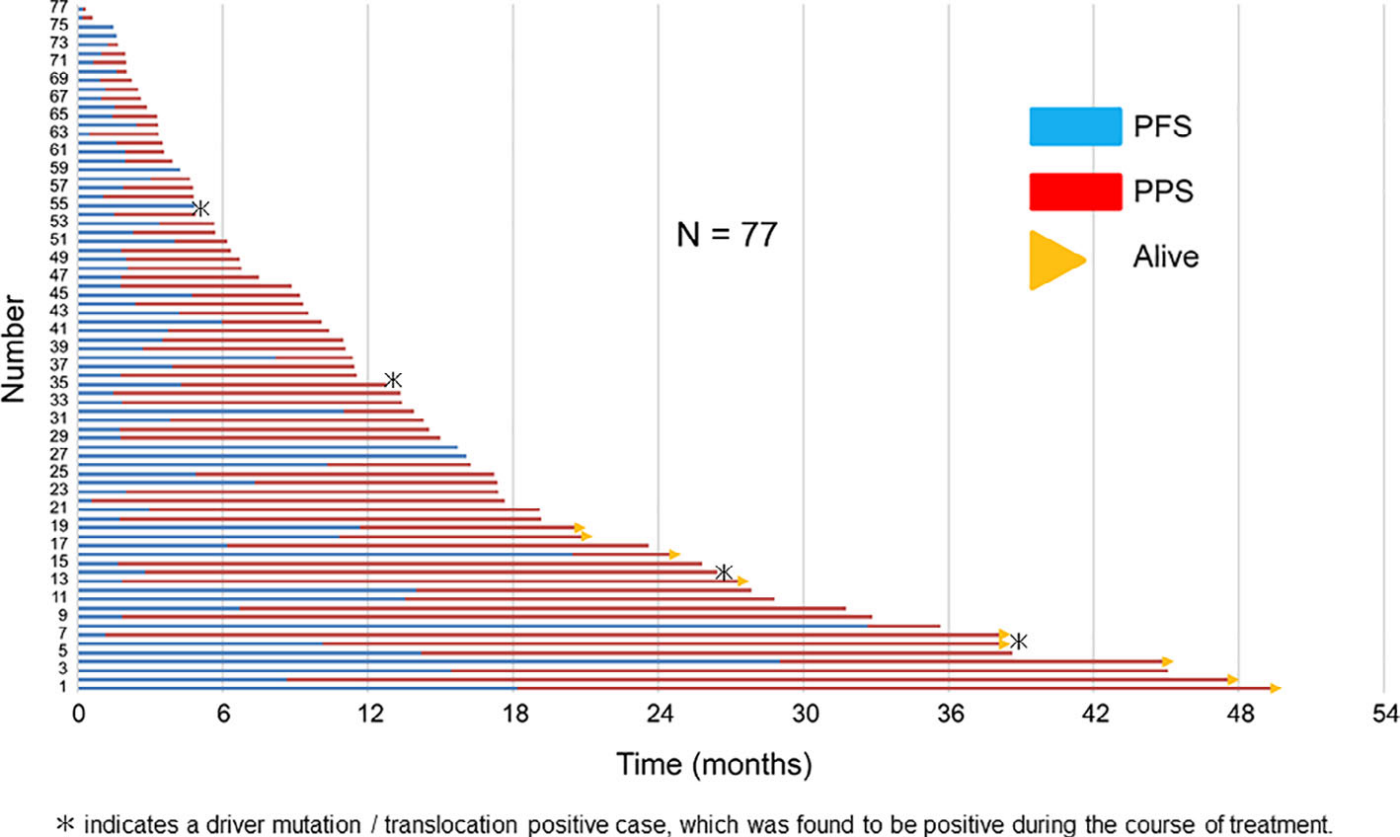
Progression‐free survival (PFS) and post‐progression survival (PPS) in the overall population

The correlations of PFS and PPS with OS are presented in Figure 3(a), (b). Linear regression and Spearman rank correlation analysis demonstrated that PPS was strongly correlated with OS (*r* = 0.85, *p* < 0.05, *R* ^*2*^ = 0.75), while PFS was moderately correlated with OS (*r* = 0.65, *p* < 0.05, *R* ^*2*^ = 0.42) (Figure 4).

### Factors related to PPS

As PPS was correlated with the OS, the relationship between the PPS and different factors was evaluated. Univariate analysis demonstrated that age at the start of second‐line treatment, performance status (PS) at the start of second‐line treatment, first‐line chemotherapy regimen (cisplatin‐based/carboplatin‐based), administration of docetaxel plus ramucirumab after nivolumab treatment, ICI rechallenge after nivolumab monotherapy, and the number of treatment regimens used post‐progression following second‐line treatment were significantly correlated with PPS (*p* < 0.05; Table 3). Multivariate analysis of these factors showed that PS at the start of second‐line treatment, ICI rechallenge after nivolumab monotherapy, and the number of treatment regimens used after the disease progression following second‐line treatment were independently associated with PPS (*p* < 0.05; Table 4).

**TABLE 3:** Univariate Cox regression analysis of baseline patient characteristics

Factors	PPS		
	HR	95% CI	*p*‐value
Sex			
Male/female	1.02	0.59–1.87	0.94
Age at the beginning of second‐line treatment (years)	1.02	1.00–1.05	0.04^*^
PS at the beginning of second‐line treatment	1.91	1.42–2.54	<0.0001^***^
Histology			
Adenocarcinoma/non‐adenocarcinoma	0.62	0.38–1.02	0.06
Stage at the beginning of second‐line treatment			
III–IV/postoperative recurrence	1.16	0.64–2.28	0.63
First‐line chemotherapy regimen			
CDDP‐based/CBDCA‐based	0.47	0.23–0.87	0.016^*^
With bevacizumab/without bevacizumab	0.87	0.49–1.48	0.63
PFS less than 3 months for nivolumab			
Yes/no	1.53	0.94–2.52	0.08
Palliative radiotherapy before nivolumab administration			
Yes/no	1.48	0.89–2.41	0.12
Administration of docetaxel plus ramucirumab after nivolumab treatment			
Yes/no	0.36	0.21–0.60	<0.0001^***^
ICI rechallenge after nivolumab treatment			
Yes/no	0.22	0.10–0.45	<0.0001^***^
Number of regimens after disease progression following second‐line nivolumab	0.53	0.41–0.67	<0.0001^***^

*Note*: ^*^ *p* < 0.05; ^**^ *p* < 0.01; ^***^ *p* < 0.001.

Abbreviations: CBDCA, carboplatin; CDDP, cisplatin; CI, confidence interval; HR, hazard ratio; ICI, immune checkpoint inhibitor; PFS, progression‐free survival; PPS, post‐progression survival; PS, performance status.

**TABLE 4:** Multivariate Cox regression analysis

Factors	PPS		
	HR	95% CI	*p*‐value
Age at the beginning of second‐line treatment (years)	0.98	0.95–1.02	0.55
PS at the beginning of second‐line treatment	1.45	1.07–1.98	0.016^*^
First‐line chemotherapy regimen			
CDDP‐based/CBDCA‐based	0.49	0.22–1.08	0.05
Administration of docetaxel plus ramucirumab after nivolumab treatment			
Yes/no	1.08	0.51–2.25	0.83
ICI rechallenge after nivolumab treatment			
Yes/no	0.33	0.14–0.71	0.004^**^
Number of regimens after disease progression following second‐line nivolumab	0.6	0.41–0.84	0.002^**^

Note: ^*^ *p* < 0.05; ^**^ *p* < 0.01.

Abbreviations: CBDCA, carboplatin; CDDP, cisplatin; CI, confidence interval; HR, hazard ratio; ICI, immune checkpoint inhibitor; PPS, post‐progression survival; PS, performance status.

**FIGURE 5:**
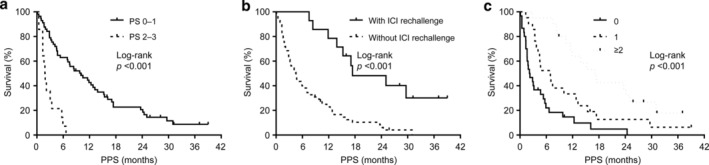
Kaplan–Meier curves of post‐progression survival (PPS) according to prognostic factors. (a) PPS according to performance status (PS) at the start of second‐line nivolumab treatment (PS 0–1: median, 10.0 months; PS 2–3: median, 1.8 months). (b) PPS according to immune checkpoint inhibitor (ICI) rechallenge after nivolumab treatment (with ICI rechallenge: median, 17.4 months; without ICI rechallenge: median, 4.5 months). (c) PPS according to the number of regimens after disease progression following second‐line nivolumab treatment (no subsequent regimen: median, 2.2 months; one regimen: median, 6.9 months; more than two regimens: median, 17.0 months)

Log‐rank tests indicated that PPS differed among patients according to PS at the start of second‐line treatment, ICI rechallenge after nivolumab monotherapy, and the number of treatment regimens used after the disease progression following second‐line treatment. According to PS at the start of second‐line treatment, the PPS in patients with PS of 0–1 and 2–3 was 10.0 and 1.8 months, respectively (log‐rank tests, *p* < 0.001; Figure 5(a)). Regarding ICI rechallenge after nivolumab treatment, the PPS in patients with and without ICI rechallenge was 17.4 and 4.5 months, respectively (log‐rank tests, *p* < 0.001; Figure 5(b)). In addition, according to the number of treatment regimens used after the disease progression following second‐line treatment, the PPS in patients who received 0, 1, and ≥2 additional regimens were 2.2, 6.9, and 17.0 months, respectively (log‐rank tests, *p* < 0.001; Figure 5(c)).

## DISCUSSION

The current analysis assessed the correlations of PFS and PPS with OS at an individual patient level in NSCLC patients who received second‐line nivolumab monotherapy. PPS was more strongly associated with OS than PFS in these patients. We identified the following independent prognostic factors for PPS: PS at the start of second‐line treatment, ICI rechallenge, and the number of treatment regimens used after disease progression following second‐line treatment. To the best of our knowledge, our study is the first attempt to evaluate individual patient‐level factors that impact PPS in NSCLC patients receiving second‐line nivolumab monotherapy.

Alternative endpoints have been examined in previous meta‐analyses.^22^, ^23^ Several different assessment criteria for confirming alternative endpoints have been suggested.^24^, ^25^ A previous study^2^ found that the duration of survival post‐progression, defined as PPS (i.e., the difference between OS and PFS), was important for assessing the validity of OS as a study endpoint. Several medical oncology studies involving patients with NSCLC^8^, ^9^, ^10^ showed that PPS was closely correlated with OS beyond first‐, second‐, and third‐line treatment. Additionally, our previous studies,^12^, ^13^, ^14^, ^15^, ^16^ based on the analysis of individual patient data, demonstrated the impact of PPS on OS in NSCLC patients who received first‐ and second‐line treatment and in advanced‐stage SCLC patients who received first‐line treatment.

This study evaluated the association between PPS and second‐line nivolumab monotherapy; the results demonstrated that PFS did not always affect OS. In addition, PFS was significantly shorter than PPS, and PPS was more strongly correlated with OS, thereby suggesting that PFS may not be a useful marker for assessing prolonged OS, and that clinical trials should instead consider factors that may influence PPS. Although PPS was more closely associated with OS in the present study, PFS was also significantly correlated with OS, thereby indicating that PFS can be a surrogate endpoint for OS in clinical trials involving patients receiving second‐line nivolumab treatment. However, in oncology trial cohorts, where a shorter PFS is expected beyond second‐line treatment, such as in patients treated with second‐line nivolumab monotherapy, it is important that factors that potentially affect PPS are regulated. Although instead of PFS we need a new surrogate endpoint or marker for clinical trials, clinical researchers should make a paradigm shift, in the way of thinking about setting endpoints, and in the development of cancer drug therapies. In particular, as ICIs have become widely used in cancer treatment, there is an urgent need to search for markers that can more accurately predict the effects of ICIs.

Based on clinical trial data of advanced NSCLC patients receiving first‐line treatment, a prolonged PPS has been reported to be correlated with good PS, first‐line monotherapy, and molecular targeted drug administration.^8^ Moreover, according to individual patient data on second‐line treatment, factors affecting PPS included the best response to third‐line chemotherapy and the number of treatment regimens used post‐progression following second‐line treatment.^15^ Further, in a study based on individual patient data on second‐line docetaxel monotherapy,^16^ factors influencing PPS included PS at the end of second‐line treatment and the number of treatment regimens used post‐progression following second‐line treatment. However, in the above‐mentioned clinical studies, nivolumab monotherapy was not administered as a second‐line treatment. In this study, factors influencing PPS included PS at the start of second‐line nivolumab monotherapy, ICI rechallenge, and the number of treatment regimens used post‐progression following second‐line nivolumab monotherapy. This study demonstrated that PS at the start of second‐line nivolumab monotherapy in advanced NSCLC patients may affect subsequent treatment, including ICI rechallenge, thereby contributing to the increase in PPS and a corresponding increase in OS. A greater number of therapeutic regimens administered after disease progression, following second‐line nivolumab treatment, is a consequence of the increasing availability of different active drugs for the subsequent‐line treatment in patients with NSCLC. Therapeutic regimens included docetaxel, pemetrexed, S‐1, docetaxel plus ramucirumab, and ICI rechallenge (nivolumab, pembrolizumab, and atezolizumab), all of which were used to some extent in our study. In this study, several different drugs were administered to treat the patients (Table 2). Ramucirumab, in combination with docetaxel, has been used for the subsequent‐line treatment of advanced NSCLC,^26^, ^27^ and could be more effective than docetaxel alone, except in patients in whom it is contraindicated. According to a previous study,^28^ a higher response was attained when docetaxel plus ramucirumab was administered for disease progression in patients with previous nivolumab usage than in those without prior nivolumab usage. Here, the use of docetaxel plus ramucirumab significantly affected PPS in univariate analysis, but not in multivariate analysis. In contrast, ICI rechallenge was found to be an independent prognostic factor for PPS in multivariate analysis. Little is known regarding the effectiveness of ICI rechallenge for disease progression. ICI rechallenge is controversial in lung cancer treatment.^29^, ^30^ A previous study^31^ suggested that the treatment outcome after readministration of programmed cell death‐1 inhibitor, following first‐line treatment with nivolumab, was significantly improved in patients with a longer duration of initial nivolumab treatment. Under these conditions, ICI rechallenge may be effective. The significance of ICI rechallenge is not currently well understood; however, our results suggest that ICI rechallenge may have a positive effect on the duration of PPS and consequently, the duration of OS.

This study has several limitations. First, the date when tumor response was measured was determined by the treating physician, which may have contributed to variation in tumor response rates and PFS. Nevertheless, this study limitation is characteristic of all retrospective studies. Second, the sample size was relatively small. However, as a limited number of patients with NSCLC receive second‐line nivolumab monotherapy at any given facility, this limitation would be difficult to resolve, particularly as our aim was to study patients at a single center. This could produce bias, although the conclusions may still be significant once the characteristics of this bias are properly understood and accounted for.

In conclusion, PPS influences OS in NSCLC patients who receive second‐line nivolumab monotherapy. When compared with PFS, PPS was more strongly correlated with OS. Furthermore, for PPS, the following independent prognostic factors were identified: PS at the start of second‐line nivolumab monotherapy, ICI rechallenge, and the number of treatment regimens used post‐progression after a second‐line nivolumab monotherapy. Our results should be validated in larger study cohorts to determine whether they are generalizable to other patient populations. Moreover, it may be prudent to consider how to improve PPS, and thus OS, while treating patients for disease progression after a second‐line nivolumab monotherapy.

## CONFLICT OF INTEREST

None of the authors have any financial or personal relationships with other people or organizations that could inappropriately influence this work.
